# Identification of Hotspot Mutations in the N Gene of SARS-CoV-2 in Russian Clinical Samples That May Affect the Detection by Reverse Transcription-PCR

**DOI:** 10.3390/diagnostics12010147

**Published:** 2022-01-08

**Authors:** Sergei A. Kiryanov, Tatiana A. Levina, Maria V. Konopleva, Anatoly P. Suslov

**Affiliations:** 1 Federal State Budget Institution “National Research Centre for Epidemiology and Microbiology Named after Honorary Academician N.F. Gamaleya” of the Ministry of Health of the Russian Federation, 123098 Moscow, Russia; tlevina2006@gmail.com (T.A.L.); maria-konopleva@rambler.ru (M.V.K.); Suslov.Anatoly@gmail.com (A.P.S.); 2 OOO “DNA-Technology”, 117587 Moscow, Russia

**Keywords:** SARS-CoV-2, RT-PCR, *E* gene, *N* gene, polymorphism, mutation

## Abstract

Sensitive and reliable diagnostic test systems based on real-time PCR are of great importance in the fight against the ongoing SARS-CoV-2 pandemic. The genetic variability of the SARS-CoV-2 virus leads to the accumulation of mutations, some of which may affect the sensitivity of modern PCR assays. The aim of this study was to search in Russian clinical samples for new mutations in SARS-CoV-2 gene *N* that can affect the detection by RT-PCR. In this study, the polymorphisms in the regions of the target gene *N* causing failed or poor detection of the target *N* in the RT-PCR assay on 12 selected samples were detected. Sequencing the entire *N* and *E* genes in these samples along with other 195 samples that were positive for both target regions was performed. Here, we identified a number of nonsynonymous mutations and one novel deletion in the *N* gene that affected the ability to detect a target in the *N* gene as well a few mutations in the *E* gene of SARS-CoV-2 that did not affect detection. Sequencing revealed that majority of the mutations in the *N* gene were located in the variable region between positions 193 and 235 aa, inside and nearby the phosphorylated serine-rich region of the protein N. This study highlights the importance of the further characterization of the genetic variability and evolution of gene *N*, the most common target for detecting SARS-CoV-2. The use of at least two targets for detecting SARS-CoV-2, including one for the *E* gene, will be necessary for reliable diagnostics.

## 1. Introduction

The novel coronavirus disease 2019 (COVID-19) pandemic, caused by the SARS-CoV-2 virus, is still a major threat to health. In the laboratory testing for detecting SARS-CoV-2, reverse transcriptase real-time PCR (RT-qPCR) assays have played a pivotal role in combating the ongoing coronavirus disease 2019 (SARS-COVID-19) spread. Since the start of the COVID-19 outbreak, various RT-qPCR assays have been developed. Commercial assays targeting mainly SARS-CoV-2 nucleocapsid (*N*), small envelope (*E*), and *RdRp* genes have been developed [1]. 

Gene *N* is the most commonly used SARS-CoV-2 detection target for viral diagnosis [2,3]. The nucleocapsid protein (N) involved in the viral assembly, replication, and regulation of host immune response plays important roles in the viral life cycle. The N protein is 419 amino acid (aa) long and composed of three distinct domains: an N-terminal domain (NTD)/RNA-binding domain (46–176 aa), a serine/arginine-rich (SR-rich; 184–204 aa) linker region (LKR; 182–247 aa), and a C-terminal domain (CTD; 247–364 aa) [4]. The targets for the detection of the *N* gene were introduced to all three domains. Gene *N* is expressed at a high level compared with other viral protein genes after infection with SARS-CoV-2 [5], which also makes it a preferred target for the detection of SARS-CoV-2. 

The multifunctional E protein is the smallest of the structural proteins encoded by the SARS-CoV-2 genome [6]. It is abundantly expressed inside the infected cell; however, only a small portion is incorporated into the virion envelope [7,8]. E protein monomers form pentameric ion channels similar to viroporins. The highly conserved E protein possesses a C-terminal PDZ-binding motif that induces immunopathology by overexpression of inflammatory cytokines [9]. These features of the E protein play a critical role in the exacerbated immune response, causing acute respiratory syndrome, the leading cause of death in SARS-CoV-2 [10]. Therefore, the E protein appears to be an attractive target for SARS-CoV-2 detection.

The genetic variability of the SARS-CoV-2 virus leads to the accumulation of mutations in the target regions used in modern diagnostic test systems for detecting SARS-CoV-2. In particular, it was noted that the *N* gene is one of the most nonconserved genes in the SARS-CoV-2 genome, and it accumulates a larger number of mutations, whereas the gene encoded envelope protein is mostly conserved [10,11,12,13]. The *N* and *E* gene assays are recommended by the WHO for first-line screening of COVID-19 cases [14,15], and are applied in many countries, including Russia. For example, DNA-Technology (Moscow, Russia) targets the SARS-specific sequences in the *N* and *E* genes.

Here, we report the identification of a number of single-nucleotide polymorphisms (SNPs) in the *N* gene of SARS-CoV-2 and single deletion in 12 Russian isolates that were associated with the failure of the qRT-PCR that targets the *N* gene in the SARS-CoV-2 test (DNA-Technology, Moscow, Russia). As this dual-target assay also detects a region in the *E* gene, these samples were still correctly identified as SARS-CoV-2 positive. Further sequencing of the entire sequence of the *N* and *E* genes of an expanded sample of isolates collected for the period of August 2020 to March 2021 made it possible to reveal the further evolution of the *N* gene with local accumulation (hotspot) of mutations in the SR region and linker region.

## 2. Materials and Methods

### 2.1. Sample Collection and Handling

Nasopharyngeal swabs were collected from patients that were admitted to Medical Center Citilab (Moscow, Russia) with presumable SARS-CoV-2 infection between August of 2020 and March of 2021. Informed consent was obtained from all subjects involved in the study. Nasopharyngeal swabs were immersed into viral transport medium (STOR-F) immediately after sample collection. Samples were stored at 4 °C until testing.

### 2.2. Nucleic Acid Extraction

Total nucleic acid was extracted from STOR-F using the Proba-NK-S RNA/DNA extraction kit (DNA-Technology, Moscow, Russia) according to the manufacturer’s instructions. Aliquoted nucleic acid and residual samples were stored at −70 °C until further testing with RT-qPCR tests. All samples were tested after one initial freeze–thaw cycle.

### 2.3. Real-Time Quantitative RT-PCR

Real-time qPCR was performed by using the real-time thermal cycler DTprime (DNA-Technology, Moscow, Russia). Amplification and detection were carried out in 96-well plates, applying SARS-CoV-2/SARS-CoV assay (DNA-Technology, Moscow, Russia), which targets genes *E* and *N* of SARS-CoV-2 according to the manufacturer’s instructions. The reaction mix utilizes a 10 μL template and a 15 μL master mix. The detection limit of the kit is 10 copies per 25 μL reaction mix. Briefly, 10 μL of extracted RNA was added to 15 μL of Real-time One-step Buffer and 2019-nCoV Oligo Mix and 0.5 μL of Real-time One-step Enzyme. The amplification conditions consisted of an initial reverse transcription at 35 °C for 20 min, followed by PCR activation at 95 °C for 5 min, followed by 5 cycles of 10 s at 95 °C and 10 s at 64 °C and then 45 cycles of 5 s at 94 °C and 10 s at 64 °C. The result was analyzed using a cycle threshold value (Ct-value) < 40 for all target genes defined as a positive result. The negative RT-qPCR result was set as a Ct value equal to 40.

### 2.4. Sanger Sequencing

We sequenced the entire *N* gene in 207 samples by Sanger sequencing. cDNA was prepared using a SuperScript IV RT cDNA synthesis kit (Thermo Fisher Scientific Inc. (Invitrogen), Waltham, MA, USA). Gene *N* was amplified using 3 sets of primers generating amplicon lengths of about 500 nts with 50 nt overlaps. The sequencing primers of set 1 for the *N* gene are the following: CovNg-1F 5′-CATGACGTTCGTGTTGTTTTAGAT-3′, CovNg-1R 5′-GCCAATGTGATCTTTTGGTGTATT-3′. Set 2 for the *N* gene: CovNg-2F 5′-GAATACACCAAAAGATCACATTGGCA-3′, CovNg-2R 5′-TCCTTGTCTGATTAGTTCCTGGTCC-3′. Set 3 for the *N* gene: CovNg-3F 5′-AAACGTACTGCCACTAAAGCATACA-3′, CovNg-3R 5′-TTATATAGCCCATCTGCCTTGTGT-3′. Gene *E* was amplified, applying 1 set of primers as follows: CovEg-F 5′-GACTACTAGCGTGCCTTTGTAAGC-3′, CovEg-R 5′-CTGCCATGGCTAAAATTAAAGTTC-3′. Taq DNA Polymerase High Fidelity (Thermo Fisher Scientific Inc. (Invitrogen), Waltham, MA, USA) was used. The sequence of the Wuhan-Hu-1 SARS-CoV-2 isolate was used as the reference genome (NCBI GenBank NC_045512.2). Sequencing was performed using a BigDye™ Terminator v3.1 Cycle Sequencing Kit (Thermo Fisher Scientific Inc. (Invitrogen), Waltham, MA, USA). All procedures were performed according to the manufacturer’s instructions.

## 3. Results

### 3.1. PCR and Sequencing Analysis of 12Russian Isolates with the Failure of Detection in the N Gene

In total, over 9800 samples were tested using the DNA-Technology test system for the period of August 2020 to March 2021, of which 574 (5.9%) were SARS-CoV-2 positive. Among them, 12 positive samples showed conflicting results on the simultaneous detection of genes *N* and *E* on the target. Three samples were detected as positive for the *E* gene, the threshold cycle value Cp = 22.8, 25.6, and 28.2, respectively, and were not detected for *N*. In the remaining samples, the value of the threshold cycle Cp for the target N exceeded Cp for E in the range of 3.7–5.6 cycles (Table 1). Thus, it was suggested that there were possible polymorphisms in the *N* gene detection site both in the nucleotide sequences of the primers and in the area complementary to the hybridization probe.

To elucidate negative results for the *N* gene in the assay, we performed the viral target sequencing with a focus on the analysis of the *N* and *E* genes for all samples used in this study. The entire nucleotide sequence of the *N* and *E* genes of these samples was sequenced. All 12 samples belonged to the B.1.1(20GR) clade, because they carried the triple mutation GGG28881-28883AAC in the *N* gene, which did not affect the detection of the test system at the target N. The most common mutation was at position G28851C/T, resulting in the S193T/I amino acid substitution. This mutation was detected in 6 samples, including G28851C in 5 samples and G28851T in 1 sample (Table 1). Sequencing revealed that the G28851C/T mutation falls into the 3′region of the forward primer that significantly impairs the detection of the target N of the DNA-Technology test system. In 2 samples undetected for the target N, this mutation was detected together with either the C28905T (A211V) mutation or the C28905T (A211V) and G28376A (A35T) double mutations. In addition, the C28905T (A211V) mutation was detected in 2 other samples. The mutation C28905T (A211V) was mapped in the region of probe hybridization that also leads to the deterioration of detection by the target N. The most interesting was the sample with several mutations, including point mutations at positions G28690T (L139F), A28858G (R195R), G28975T (M234I), and deletion del28896–28919, causing in-frame reduction of 8 amino acid residues, A208—G215. The deletion occurred at the complementation site of the hybridization probe, causing a complete absence of detection by the target N. In addition, a single G28916A mutation causing the G215S amino acid substitution was found in 2 samples with impaired detection for the *N* gene. C28909T substitution was also found in 2 other samples, which led to a synonymous mutation in the G212 codon.

Sequencing analysis revealed the presence of mutations in all samples with impaired detection in the *N* gene, either in the 3′region of the forward primer or in the region complementary to the hybridization probe. Notably, none of these results were reported as false negative by the test platform, because the *E* gene was amplified properly.

### 3.2. Sequencing the Entire N and E Genes in 195 Clinical Samples

Sequencing of the entire *N* gene in 195 samples with adequate detection of N and E targets also revealed the presence of single nucleotide substitutions presented with different frequencies of occurrence: the most frequent mutations, G28975T (M234I), were found in 5 samples (2.6% (5/195)), C28866T (T198I) in 3 samples (1.5% (3/195)), C28887T (T205I) in 3 samples (1.5% (3/195)), G28376A (A35T) in 3 samples (1.5% (3/195)), G28655T (D128Y) in two samples (1.0% (2/195)), and C29250T (P326L) in two samples. Out of 195 samples, 152 (i.e., 77.9%) also carried the mutation GGG28881-28883AAC in the *N* gene and belonged to the B.1.1.20GR clade. Five samples were identified as B.1.1.7 strain and carried the triple mutation GGG28881-28883AAC along with the mutations GAT28280-28282CTA (D3L) and C28977T (S235F) in gene *N*. The remaining nonsynonymous and synonymous mutations localized along the entire length of the *N* gene were found in single samples and are shown in Table 2. 

A total of 57 nonsynonymous and 23 synonymous mutations were found, localized in all functional regions of the *N* gene and in disordered regions. Eight nonsynonymous mutations were mapped to the *N*-terminal domain (NTD) (46–176 aa), 11 mutations were detected in the *C*-terminal domain (CTD) (247–364 aa), and 17 mutations were detected in the serine/arginine-rich region (SRD) (184–204 aa), while 11 mutations were found in the linker region (205–246 aa).

**Figure 1:**
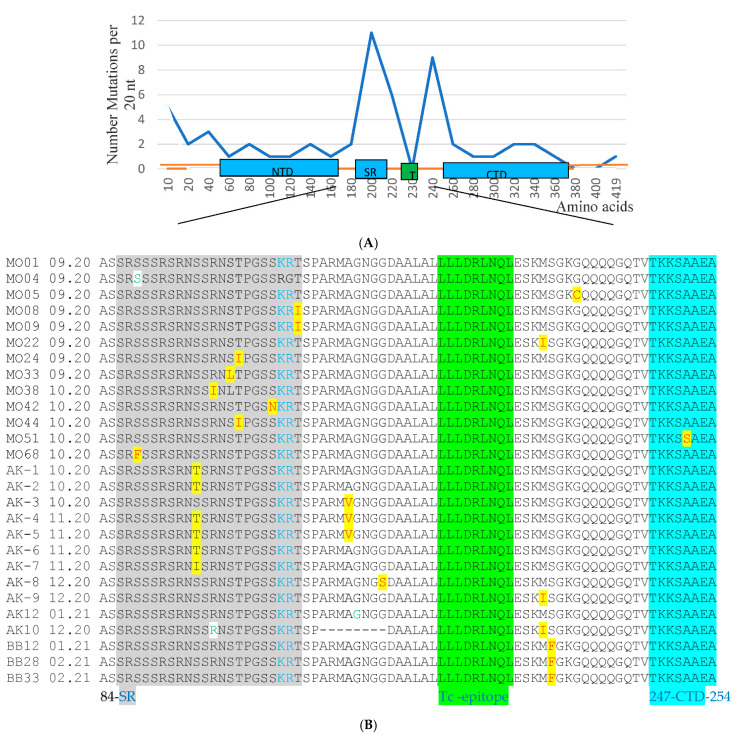
Mutation spectra of the SARS-CoV-2 nucleocapsid protein in the Russian isolates. (**A**) AA mutation distribution per 20 aa in amino acid strains through the N protein. (**B**) Local alignment of the SR-rich and linker region of the N protein with mutation mapping. NTD, N-terminal RNA binding domain; SR, serine/arginine-rich domain; CTD, C-terminal dimerization domain; Tc-epitope, predictive T-cell epitope. Nonsynonymous mutations are highlighted in yellow; synonymous mutations are highlighted in white.

Ten and 1 mutations were detected in the 5′ and 3′ disordered regions of the *N* gene, respectively. Interestingly, no mutations were found in the region of the predicted T-cell epitope between 223 and 231 aa [16]. Summarizing the representation of all detected mutations and their localization in gene *N*, it was noticed that the main local mutation density (49.1% (28/57)) in Russian isolates was observed in the region between amino acid residues S193 and D235 of gene *N* (Figure 1).

This hotspot mutation region includes both the “SR-rich region”, between amino acids 184 and 204, which contains phosphorylation sites at positions S197, S202, and T205 [17], and the flanking region of low complexity up to position D235. Interestingly, the single mutations S197L, S202N, and T205I, potential phosphorylation sites in the nucleocapsid protein, were detected in several isolates.

Sequencing of the entire *E* gene revealed the presence of nonsynonymous mutations in the 5′ region of the gene. Nucleotide substitutions C26261T, causing the S6L mutation, and G26257A (V5I) were found in 7 (3.6% (7/195)) and 4 (2.1% (4/195)) samples, respectively. Interestingly, these mutations were found mainly in the samples with mutations in the *N* gene at positions S193T/I and A211V and in the sample with the A208—G215 deletion. However, the localization of these mutations outside the region of detection by the target of the *E* gene did not affect the detecting ability of qPCR.

## 4. Discussion

It was previously noted that the genetic variability of the SARS-CoV-2 virus leads to the accumulation of mutations, including the regions of the main target genes, *N* and *E*, used in modern diagnostic test systems for detecting SARS-CoV-2 [18,19,20]. In particular, it was noted that the *N* gene, the most commonly used SARS-CoV-2 detection target, is one of the most non-conserved genes in the SARS-CoV-2 genome [21]. Sequencing of the entire *N* and *E* genes of SARS-CoV-2 from 12 isolates of SARS-CoV-2 from Moscow and the Moscow Region, which showed dubious signals or failure of detection for the *N* gene by testing with the SARS-CoV-2 assay (DNA-Technology, Moscow, Russia), revealed the presence of mutations both in the complementation regions with the forward primer and in the hybridization probe in the *N* gene, but not in the *E* gene.

Our data confirm that in places with a high level of SARS-CoV-2 infection rate, such as Moscow and the Moscow Region, and due to the high transmissibility of the virus and the spread of novel variants with mutations in a primer or probe location, the effectiveness of RT-PCR could be impaired. Several mutations in the binding sites of the target primers or probes in the *N* gene of SARS-CoV-2/SARS-CoV in almost all used diagnostic test systems for SARS-CoV-2 with the detection of gene *N* have been reported [13,22,23,24]. That leads to a decrease in the sensitivity of the assay and potentially underestimated diagnostics if only one detection target is used. To help prevent false-negative results in the diagnostic assay due to point mutations or deletions/insertions, continuous mismatch monitoring by sequencing and routine use of at least two SARS-CoV-2 targets should be performed.

Sequencing of 195 SARS-CoV-2 samples with adequate detection of targets in genes *N* and *E* collected from August 2020 to March 2021 revealed a number of additional mutations, their frequency and localization, and further evolution of the *N* gene in clade B.1.1. isolates with the double mutation R203K and G204R.

Although mutations were found through the entire length and in all structural domains in the *N* gene, the main accumulation of nonsynonymous mutations (49.1% (28/57)) in Russian isolates occurred in the region between amino acid residues S193 and S235 of the N protein. The most common mutations in the N (S193T/I, T198I, T205I, A211V, G215S) and the del28896—28919 (A208—G215) deletion identified in this study are located within the SR-rich region (184-204 aa) and part of the intrinsically unstructured linker region. This mutation-enriched region overlaps a predicted B-cell epitope, suggesting positive selection for immune system avoidance as reported [25]. Besides nonsynonymous mutations in the previously identified phosphorylation sites, S197, S202, and T205 were found in several isolates [26]. Another region with the highest mutation density (M234I, S235F, G238C) was found within the unstructured linker region of the nucleocapsid protein and adjoining the CTD domain. Another region with a significant mutation density was the unstructured region between 3 and 35 aa, where mutations in positions A35T, P13L, and D3L were found. The latter mutation along with S235F was detected in five isolates identified as the British strain B.1.1.7.

The detection of mutations in the two indicated regions of the *N* gene (5′ unstructured and linker) and their accumulation in Russian isolates reflects further evolution of the B.1.1 lineage with the formation of new variants, such as the recently reported B.1.1.317 (N: A211V) and B.1.1.397 (N: M234I) [27] and a novel variant (N: S193T and A35T).

The majority of the mutations in gene *N* identified in this study were not unique and were found to belong to lineages of clade B.1.1 of various geographic origins [12]. However, the detected mutation patterns are characteristic of the B.1.1 lineages of the Russian origin. 

Another feature was the identification a short sequence 221–231 aa in the linker between the regions with a high mutation density, which was still intact to mutation. This region presumably corresponds to the sequence of the predicted T-cell epitope (222–231 aa) [16], which suggests so far the lack of possible escape mutations that cause escape from CD8 T-cell response.

Here, a reported deletion of 24 nucleotides causing in-frame loss of eight amino acid residues (A208—G215) was unique and the only one among the samples of Russian isolates. Similar deletions of 12 nucleotides in size with a loss of four amino acid residues (206–210 aa) are rare, as previously reported in six American isolates [28]. The deletion affects one of the two predicted in silico motifs of the conserved N-myristoylation site (GDAAL). This modification of viral proteins causes their localization in areas of the cell membrane by binding to lipid fragments with the N-terminal glycine site [29]. The occurring *N* gene deletion may have a significant impact on the interaction of the nucleocapsid protein and host proteins and viral pathogenesis.

Our results imply continuous evolution of the SARS-CoV-2 *N* gene with accumulation of mutations mainly in two regions, namely, in the linker region and the unstructured region 5′of the *N*. 

Our study has some limitations. First, a functional study of the identified point and deletion mutations was not performed. Second, epidemiological information of all SARS-CoV-2 samples collected in Moscow and the Moscow Region and used in this study was lacking. Third, the number of isolates was limited, and sequencing data for genes *N* and *E* were obtained to compare with samples that showed questionable signals or no detection of the gene *N*. For these reasons, studies that further investigate the frequency of the novel mutations in Russia and worldwide will be of practical importance for the accurate diagnosis of SARS-CoV-2.

## 5. Conclusions

Genetic variability and further evolution of the *N* gene with possible accumulation of mutations in certain regions, primarily in the unstructured linker region and the 5′region of the gene, may cause some false-negative results. The emergence of novel SARS-CoV-2 variants with novel mutations, particularly in gene *N*, may deteriorate detection by RT-PCR assays based on a single gene target. The use of at least two targets for the detection of SARS-CoV-2, including the more conserved gene *E*, provides higher diagnostic sensitivity. Our findings may also help to better understand viral pathogenesis and the evolution of individual viral genes during the current COVID-19 pandemic.

## Figures and Tables

**Table 1:** Summary of RT-PCR assay and sequencing data obtained from the samples with polymorphisms in the *N* gene-affected *N* gene detection.

Sample	Test Data	RT-PCR, Cp		Detected Mutations *			
		Gene *E*	Gene *N*	Gene *E*		Gene *N*	
				Nucleotide	Amino Acid	Nucleotide	Amino Acid
AK-1	06.10.20	27.0	31.7	C26261T	S6L	G28851C C28909T	S193T G212G
AK-2	06.10.20	25.8	29.6	G26257A	V5I	G28851C	S193T
AK-3	08.10.20	26.1	29.9	C26261T	S6L	C28905T	A211V
AK-4	06.11.20	25.6	n/d **	n/d	n/d	G28851C C28905T	S193T A211V
AK-5	06.11.20	24.9	n/d	n/d	n/d	G28376A G28851C C28905T	A35T S193T A211V
AK-6	06.11.20	28.2	33.8	C26261T	S6L	G28851C C28909T	S193T G212G
AK-7	06.11.20	27.3	31.5	n/d	n/d	G28851T	S193I
AK-8	12.01.21	25.7	29.6	n/d	n/d	G28916A	G215S
AK-16	10.11.20	26.4	30.5	n/d	n/d	C28905T	A211V
AK-10	24.12.20	24.0	n/d	C26261T	S6L	del 28896-28919 G28690T A28858G G28975T	A208-G215 L139F R195 M234I
AK-11	12.01.21	25.3	29.6	n/d	n/d	G28916A	G215S
AK-12	15.02.21	27.8	31.7	n/d	n/d	C28909T	G212G

* Nonsynonymous and synonymous mutations in genes *N* and *E*; ** n/d—no change detected.

**Table 2:** Summary of sequencing data obtained from the Russian samples with polymorphisms in the *E* and *N* genes.

Sample	Test Data	Detected Mutations				Gene *N* Region *
		Gene *E*		Gene *N*		
		Nucleotide	Amino Acid	Nucleotide	Amino Acid	
M002	06.09.20	n/d **	n/d	C28866T	T198I	SR
M005	07.09.20	n/d	n/d	G28985T	G238C	LKR
M008	12.09.20	n/d	n/d	C28887T	T205I	LKR
M009	12.09.20	n/d	n/d	C28887T	T205I	LKR
M022	15.09.20	n/d	n/d	G28975T	M234I	LKR
M024	18.09.20	n/d	n/d	C28866T	T198I	SR
M033	22.09.20	C26261T	S6L	C28863T	S197L	SR
M038	03.10.20	n/d	n/d	G28857T	R195I	SR
M042	09.10.20	n/d	n/d	G28878A	S202N	SR
M044	09.10.20	n/d	n/d	C28866T	T198I	SR
M047	09.10.20	n/d	n/d	G28655T	D128Y	NTD
M051	11.10.20	n/d	n/d	G29024T	A251S	CTD
M068	15.10.20	n/d	n/d	C28830T	S186F	SR
M071	15.10.20	n/d	n/d	G28655T	D128Y	NTD
AK-9	09.12.20	n/d	n/d	G28975T	M234I	LKR
AK-18	11.12.20	C26261T	S6L	G28376A	A35T	5′DLR
AK-23	11.12.20	n/d	n/d	G28975T C28651T	M234I N126	LKR NTD
AK-26	11.12.20	G26257A	V5I	C29095T C29218T	F274 F315	CTD CTD
AK-31	14.12.20	n/d	n/d	G28975T	M234I	LKR
AK-32	14.12.20	n/d	n/d	G28655T	D128Y	NTD
AK-37	14.12.20	n/d	n/d	G28975T	M234I	LKR
AK-39	14.12.20	n/d	n/d	C28697T	P142S	NTD
AK-41	15.12.20	n/d	n/d	C28887T G28195T	T205I N140K	LKR NTD
AK-46	17.12.20	n/d	n/d	G28985T	G238C	LKR
AK-49	17.12.20	n/d	n/d	G29024T	A251S	CTD
AK-56	18.12.20	n/d	n/d	G28878A	S202N	SR
AK-57	18.12.20	n/d	n/d	G28376A	A35T	5′DLR
AK-72	14.01.21	n/d	n/d	C28755T	L161F	NTD
AK-77	14.01.21	n/d	n/d	C28775T	P168S	NTD
AK-81	14.01.21	n/d	n/d	C28311T	P13L	5′DLR
AK-84	14.01.21	n/d	n/d	C29250T	P326L	CTD
AK-85	14.01.21	n/d	n/d	C29153A	Q294K	CTD
AK-92	15.01.21	n/d	n/d	G29315C	D348H	CTD
AK-97	15.01.21	n/d	n/d	C28826T C29463T	R185C A397V	SR 3′DLR
AK-104	15.01.21	n/d	n/d	C29067T	T265I	CTD
AK-113	16.01.21	n/d	n/d	G28376A	A35T	5′DLR
AK-118	16.01.21	n/d	n/d	C29095T	F274F	CTD
AK-124	16.01.21	n/d	n/d	G29148C	I292T	CTD
AK-135	16.01.21	n/d	n/d	C29250T	P326L	CTD
AK-143	16.01.21	n/d	n/d	C29149T	I292I	CTD
BB ***-12	22.01.21	n/d	n/d	28280GAT>CTA C28977T	D3L S235F	5′DLR LKR
BB-28	26.02.21	n/d	n/d	28280GAT>CTA C28977T	D3L S235F	5′DLR LKR
BB-31	26.02.21	n/d	n/d	28280GAT>CTA C28977T	D3L S235F	5′DLR LKR
BB-37	26.02.21	n/d	n/d	28280GAT>CTA C28977T	D3L S235F	5′DLR LKR
BB-37	03.03.21	n/d	n/d	28280GAT>CTA C28977T	D3L S235F	5′DLR LKR

* NTD—N-terminal domain, CTD—C-terminal domain, SR—serine/arginine-rich region, LKR—unstructured linker region, 5′DLR and 3′DLR—5′- and 3′-disordered regions of *N* gene; ** n/d—no change detected; *** BB—strain B.1.1.7.

## Data Availability

Not applicable.
